# Expression of a secretory α-glucosidase II from *Apis cerana indica* in *Pichia pastoris* and its characterization

**DOI:** 10.1186/1472-6750-13-16

**Published:** 2013-02-18

**Authors:** Jirattikarn Kaewmuangmoon, Manlika Kilaso, Ubolsree Leartsakulpanich, Kiyoshi Kimura, Atsuo Kimura, Chanpen Chanchao

**Affiliations:** 1 Program in Biotechnology, Faculty of Science, Chulalongkorn University, 254 Phayathai Road, Bangkok, 10330, Thailand; 2 National Center for Genetic Engineering and Biotechnology, National Science and Technology Development Agency, 113 Thailand Science Park, Phaholyothin Road, Pathumthani, 12120, Thailand; 3 Honeybee Research Group, National Institute of Livestock and Grassland Science, Ibaraki, 305-0901, Japan; 4 Division of Applied Bioscience, Graduate School of Agriculture, Hokkaido University, Sapporo, 060-8589, Japan; 5 Department of Biology, Faculty of Science, Chulalongkorn University, 254 Phayathai Road, Bangkok, 10330, Thailand

**Keywords:** Apis cerana indica, α–glucosidase, Expression, Homology, Recombinant enzyme

## Abstract

**Background:**

α–glucosidase (HBGase) plays a key role in hydrolyzing α-glucosidic linkages. In *Apis mellifera*, three isoforms of HBGase (I, II and III) have been reported, which differ in their nucleotide composition, encoding amino acid sequences and enzyme kinetics. Recombinant (r)HBGase II from *A. cerana indica* (r*Aci*HBGase II) was focused upon here due to the fact it is a native and economic honeybee species in Thailand. The data is compared to the two other isoforms, *Aci*HBGase I and III from the same bee species and to the three isoforms (HBGase I, II and III) in different bee species where available.

**Results:**

The highest transcript expression level of *AciHBGase II* was found in larvae and pupae, with lower levels in the eggs of *A. cerana indica* but it was not found in foragers. The full-length *AciHBGase II* cDNA, and the predicted amino acid sequence it encodes were 1,740 bp and 579 residues, respectively. The cDNA sequence was 90% identical to that from the HBGase II from the closely related *A. cerana japonica* (GenBank accession # NM_FJ752630.1). The full length cDNA was directionally cloned into the pPICZαA expression vector in frame with a (His)_6_ encoding C terminal tag using *Eco*RI and *Kpn*I compatible ends, and transformed into *Pichia pastoris*. Maximal expression of the r*Aci*HBGase II–(His)_6_ protein was induced by 0.5% (v/v) methanol for 96 h and secreted into the culture media. The partially purified enzyme was found to have optimal α-glucosidase activity at pH 3.5 and 45°C, with > 80% activity between pH 3.5–5.0 and 40–55°C, and was stabile (> 80% activity) at pH 4–8 and at < 25–65°C. The optimal substrate was sucrose.

**Conclusions:**

Like in *A. mellifera*, there are three isoforms of *Aci*HBGase (I, II and III) that differ in their transcript expression pattern, nucleotide sequences and optimal enzyme conditions and kinetics.

## Background

α–Glucosidases belong to the glycosyl hydrolase family (EC 3.2.1.20, HBGase) and catalyze the hydrolysis of non reducing terminals of substrates, such as sugars like sucrose and maltose and other glucosides including phenyl α–glucoside, to liberate α-glucose [1]. Based on their primary structure (amino acid sequences), members of Family 13 (GH13) have four regions that are important in their catalytic action [2,3]. In contrast, HBGase II enzymes do not have these four regions and belong to members of Family 31 (GH31) [4]. According to X-ray crystallographic analysis, the tertiary structures of both HBGase I and II enzymes from the bacteria *Bacillus cereus* and *Sulfolobus sulphataricus*, respectively, have a (β/α)_8_ barrel structure as the catalytic domain but they differ in the features of the active sites involved in the catalytic reactions [5,6].

Three isoforms of HBGase have been reported in *A. mellifera* (*Am*HBGase I, II and III), which differ in their substrate specificity, mass, nucleotide and predicted amino acid sequences, and expression patterns in different tissues and developmental stages in the insect [7,8]. Moreover, these three enzymes showed different pH and temperature optima for enzyme activity, as well as pH and thermal stabilities, and sugar substrate preferences [9-11].

Homologs of these three *Am*HBGase isoforms (I, II and type II-like HBGases) have also been reported in the yeast *Sporothrix schenckii*, where they were found to be located in the endoplasmic reticulum and to be involved in processing of the *N*-glycan core for glycoprotein biosynthesis [12]. However, these three yeast homologs differ in their molecular mass and biochemical characters.

In the biotechnology industry, HBGases are key enzymes for the preparation of alcoholic beverages and brewing, such as in the wine and sake industry. Indeed, the HBGase in rice (*Oryza sativa*) is directly involved in the alcohol fermentation process in sake processing. Thus, Iwata et al. [13] purified the HBGase from *O. sativa* Yamadanishiki and reported that the enzyme had a different substrate specificity (nigerose) from the HBGase I (maltotriose and maltotetraose) purified from *O. sativa* cv. Shinsetsu, and also hydrolyzed nigerose and kojibiose better than the HBGase II from *O. sativa* cv. Shinsetsu.

Furthermore, Michlmayr et al. [14] reported that the aroma of wine was in part due to the action of glycosidases from *Oenococcus oeni*, a wine-related lactic acid bacterium, since the precursors of the volatile monoterpene based constituents (the primary grape aroma) were monoglucosides and diglucosides that were cleaved by the HBGase releasing the volatile monoterpenes.

Other than catalyzing the cleavage of α–glucosyl residue substrates, HBGase can also catalyze transglycosylation reactions to synthesize various α-glucosylated compounds. Accordingly, this enzyme-catalyzed transglycosylation is used in the biosynthesis of carbohydrates that are important for humans [15]. Zhou et al. [16] purified a novel extracellular α–glucosidase II (210 kDa) with a high transglycosylation activity from *Arthrobacter* sp. DL001. This enzyme could transfer glucosyl groups from donors containing an α-(1,4)-glucosidic bond specific to glucosides, xylosides and alkyl alcohols in α-(1,4)- or α(-1,6)-linkages.

Thus, it is of relevance to find new sources of HBGases with different enzyme properties, like the conformational stability to heat, pH and denaturants, a wide range of or different specific substrate specificities, catalysis of transglucosylation reactions, and enzymatic synthesis of novel oligosaccharides, so as to better fit the specific requirements needed by each respective application. Accordingly, HBGase enzymes have been purified from many organisms, but especially from microorganisms [17]. Cihan et al. [17] successfully purified HBGases from *Geobacillus toebii* strain E134, isolated from a hot spring. The intracellular HBGase showed an optimal enzyme activity at 65°C and pH 7.0, while the extracellular one showed an optimal activity at 70°C and pH 6.8. Interestingly, both enzymes were active over the temperature and pH ranges of 35–70°C and 4.5–11.0, respectively.

Recently, in order to supply sufficient HBGase for industrial production, not only native HBGase from new sources has been explored, but recombinant (r)HBGase has gained in interest. Heterogeneous expression of rHBGase in the *Pichia pastoris* expression system has been widely used since it achieves a low production cost and highly efficient production, whilst maintaining glycosylation and correct folding, and is free of potential bacterial endotoxins and lipopolysaccharides unlike bacterial expression systems. Konishi et al. [18] successfully synthesized novel artificial glycolipids using the rHBGase from *Geobacillus* sp. HTA-462, originally isolated from a deep sea sediment. The mimic glycolipids produced could be used as biosurfactants and materials in a diverse array of biological applications by changes to their physicochemical properties.

In this research, the transcript expression pattern and sequence determination of the *HBGase II* from *Apis cerana indica* (*AciHBGase II*) together with the expression, purification and characterization of the r*Aci*HBGase II enzyme (as a C-terminal (His)_6_ tagged chimera) activity was evaluated. To our knowledge, this is the first report of the *Aci*HBGase gene and recombinant enzyme from this Thai native and economic honeybee species, although the native form has not yet been purified and characterized. The outcome from this research is a potential new source of HBGase II that may be applied in the glucose related industries.

## Methods

### Sample collection

*A. cerana indica* were collected from an apiary in Samut Songkram province. Eggs, larvae and pupae were collected directly from hives while foragers were collected from the returning flight at the entrance area of the hive. Samples were kept at -80°C until used.

### RNA extraction

Samples (eggs, larvae, pupae and forager bees) were taken from -80°C storage and were separately ground with a pestle in a mortar under liquid nitrogen and then total RNA was extracted from the ground samples using a standard acid-guanidine thiocyanate-phenol-chloroform method [19]. The quality of RNA was visually assayed under UV-transillumination after resolution by 1.2% (w/v) formaldehyde/ agarose gel electrophoresis and ethidium bromide (EtBr) staining. After that, poly A^+^ mRNA was isolated using the oligotex (dT)_30_ super kit (catalog # 9086, Takara) as per the manufacturer’s instructions. To monitor the RNA concentration and its relative purity, the absorbance at 260 and 280 nm was measured.

### Transcript expression pattern and sequence analysis of *AciHBGase II*

Primer design was based on the cDNA sequence of *AmHBGase II* (GenBank accession # NM_001040259). All primers (Table 1) were designed using the Primer 3 program (http://frodo.wi.mit.edu/primer3/) and manually checked. In addition, as a template control, a 350 bp fragment of the *28S rDNA* gene was amplified using the primers shown in Table 1. RT-PCR was performed using an Access RT-PCR system kit (catalog # A1250, Promega) as per the supplier’s instructions, and reactions without the RNA template or without reverse transcriptase were used as negative controls. For analysis of the *AciHBGase II* transcript expression pattern, the extracted total RNA from eggs, larvae, pupae and foragers was used, while for sequence analysis the total RNA extracted from the pupae was used as the RT-PCR template. The reaction mixture (25 μl final volume) was comprised of 1 x AMV / *Tfl* reaction buffer, 0.2 μM of each dNTP, 0.4 μM of each forward (F) and reverse (R) primer, 1 mM MgSO_4_, 0.1 units (U) of AMV reverse transcriptase, 0.1 U *Tfl* DNA polymerase and 200 ng of RNA template. All RT-PCR reactions were performed under previously optimized conditions as follows: 1 cycle of 48°C for 45 min and 94°C for 2 min; 30 cycles of 94°C for 30 s, Ta°C (see Table 1) for 30 s, and 68°C for 2 min; and finally 1 cycle of 68°C for 7 min. RT-PCR products were resolved by 1.2% (w/v) agarose-TBE gel electrophoresis and visualized by UV-transillumination after EtBr staining. After that, they were purified using a QIAquick PCR purification kit (catalog # 28104, Qiagen) as per the manufacturer’s protocol and then direct sequenced commercially at the Bioservice Unit (BSU), National Science and Technology Development Agency (NSTDA), Bangkok, Thailand, using the same F and R primers (separate reactions). The obtained consensus sequences were searched against the NCBI GenBank data base for homologous sequences using the MegaBLASTn algorithm.

**Table 1:** **Primers used for the RT-PCR to obtain the full-length cDNA of *AciHBGase II***

**Primer name**	**Forward primer (5**^**′**^ → **3**^**′**^**)**	**Reverse primer (5**^**′**^ → **3**^**′**^**)**	**Ta (**^**o**^**C)**^**a**^	**Size (bp)**^**b**^
Pair 3_HBGase I	AATGGCGAGAATTTTGTGGAC	TGGAGTTTACGCTGCTTGTG	53	783
Pair 1_HBGase II	ATGTTTCGAGCGACGATAGTTAC	AAGACGCTGAGCCAATTGTT	52	503
Pair 2_HBGase II	CGAGGAGTTTCCAAGACAGC	CTCGAACATGTGGTTGATGG	42	581
Pair 3_HBGase II	AGTACTACGTGTGGCGGGAC	GGACTTGAACGCCACGTAAT	52	983
Pair 4_HBGase II	CGTGATGCTGACGTTGACTT	TTACAACCAGTCTACACCTTGCC	48	634
28S rDNA	AAAGATCGAATGGGGATATTC	CACCGGGTCCGTACCTCC	44	350

^a^Ta = annealing temperature used in the RT-PCR reaction.

^b^Size = expected amplicon size.

### Construction of the r*Aci*HBGase II-(His)_6_ encoding expression vector for *P. pastoris*

In order to amplify the full-length *AciHBGase II* cDNA the F and R PCR primers were designed to encompass the 5^′^ and 3^′^ outermost regions of the *AciHBGase II*, based upon the sequences for the related *Am*HBGase II (*HBGase II*F (5^′^ CAAAATGGAATTCTTTCGAGCGACGATAGTTA 3^′^) and *AmHBGase II*R (5^′^ CGAGGTACCCAACCAGTCTACACCTTGCC 3^′^). Note that to facilitate the directional cloning the F and R primers contained 5^′^ flanking extension sequences to yield *Eco*RI and *Kpn*I restriction sites (underlined), respectively. The RT-PCR reaction was performed under previously optimized conditions as follows: 1 cycle of 94°C for 2 min; 30 cycles of 94°C for 30 s, 52°C for 30 s and 68°C for 2 min; and finally 1 cycle of 68°C for 7 min. The expected RT-PCR product was observed by UV transillumination after resolution through a 1% (w/v) agarose-TBE gel and EtBr staining. The desired RT-PCR product was isolated and ligated into pPICZαA (Invitrogen), which had been separately digested by *Eco*RI and *Kpn*I at 37°C overnight, using a 3:1 (w/w) ratio of PCR product: vector and 1 U T4 ligase in 1 × T4 ligase buffer (6 μl total volume) at 16°C overnight, and then transformed to *P. pastoris*. Note that this construct places an in-frame C-terminal (His)_6_ tag onto the encoded protein, so in this case a r*Aci*HBGase II-(His)_6_ chimeric enzyme is encoded for.

### Transformation of *P. pastoris*

Before transformation, 5–10 μg of the recombinant plasmid was digested by *Sac*I at 37°C for 1 h. *P. pastoris* GS115 (His) strain (Invitrogen) was prepared following the protocol of the EasySelect*Pichia* expression kit (catalog # K1740-01, Invitrogen). The *Sac*I-linearized plasmid was transformed into *P. pastoris* by electroporation using Gene Pulser (Bio-Rad), as per the recommendation of the Invitrogen manual (Methods for the expression of recombinant protein in *P. pastoris*). The electroporated yeast were spread onto YPDS plates (YPD (1% (w/v) yeast extract, 2% (w/v) peptone, 2% (w/v) dextrose and 2% (w/v) agar,) with 1 M sorbitol) containing 100 μg/ml Zeocin and incubated at 30°C for 3 - 10 days until colonies formed. The His autotrophic transformants (His^+^) were selected and were retained on a YPD agar plate for further study. As a negative control, transformation was also performed using only the *sac*I linearized empty pPICZαA vector.

### Expression of the r*Aci*HBGase II-(His)_6_ enzyme

After a few transformants were selected to see the level of expression, a single transformed *P. pastoris* colony presenting the highest expressed enzyme level was inoculated into 25 ml of BMGY medium (1% (w/v) yeast extract, 2% (w/v) peptone, 100 mM potassium phosphate buffer (pH 6.0), 1.34% (w/v) yeast nitrogen base, 4 μg/ml D-biotin and 1% (v/v) glycerol). To form the inoculum, the culture was grown at 30°C, 200 rpm until the O.D. at 600 nm reached 2-6. Cells, collected by centrifugation at 704 × g, room temperature (RT) for 5 min, were then transferred into 50 ml of BMMY medium (BMGY except with methanol in place of glycerol) containing various percentages of methanol (0–10% (v/v)), and this amount of methanol was added every 24 h for various times of incubation (0–144 h). At the indicated time point, 1 ml of the induced culture was collected by centrifugation (13,226 × g, RT, 3 min) and the pellet and supernatant were stored separately at -20°C until assayed. Protein expression was determined by resolution through a reducing sodium dodecyl sulfate-polyacrylamide gel electrophoresis (SDS-PAGE) (8% (w/v) acrylamide resolving gel) and visualized by Coomassie blue staining. The protein concentration was calculated by the absorbance at 280 nm and Bradford’s assay [20].

### Partial purification of the r*Aci*HBGase II-(His)_6_ enzyme

The transformants were first cultured in 25 ml of BMGY medium at 30°C for 24 h with agitation at 200 × rpm to form the inoculum, and then the induction of r*Aci*HBgase II-(His)_6_ was performed by the addition of the optimal level of methanol (% (v/v) every 24 h determined as above) in 1.2 liter of BMMY medium for 96 h. The sample was collected by centrifugation (5,009 × g, 4°C, 10 min), and then 100 ml of the supernatant was concentrated (~100-fold) through a 10 kDa molecular weight cut off membrane (Vivaspin 20; catalog# 28-9331-02 AB, GE Healthcare) to ~1 ml. The sample composition was adjusted to that of the binding buffer (20 mM sodium phosphate pH 7.4, 0.5 M NaCl, 20 mM imidazole) and applied (200 μl at a time) sequentially to a HisTrap affinity column (1 ml in size, GE Healthcare). After that, 15 ml of binding buffer was used to wash the column and then the r*Aci*HBGase II-(His)_6_ protein was eluted with 5 ml of elution buffer (binding buffer except with 150 mM imidazole). Fractions (1 ml) were collected, mixed with 1 × protease inhibitor cocktails (Amresgo) and stored at 4°C. The apparent homogeneity of the r*Aci*HBGase II-(His)_6_ protein was evaluated by resolution of 1 μg of sample per track of a reducing SDS-PAGE (8% (w/v) acrylamide resolving gel). Renaturation and α-glucosidase activity staining (zymogen) within resolved SDS-PAGE gels was performed as reported previously [21].

### Assay for r*Aci*HBGase II-(His)_6_ enzyme activity

The α-glucosidase activity was determined using *p*-nitrophenyl α-D-glucoside (PNPG) as the substrate. The premix (0.1 ml of 0.1 M sodium phosphate buffer (pH 5.5), 0.05 ml of d-H_2_O and 0.05 ml of 5 mM PNPG) was incubated at 37°C for 10 min and then 0.05 ml of the test sample (e.g. column chromatography fraction or the r*Aci*HBGase II-(His)_6_ preparation) was added and incubated at 37°C for 10 min. The reaction was then stopped by adding 0.5 ml of 1 M Na_2_CO_3_. The control reaction was performed as above but with the addition of the sample buffer only and not the test sample (enzyme). The absorbance at 400 nm was monitored so as to measure the release of the yellow *p*-nitrophenol. One U of HBGase II activity was defined as that which liberates 1 μM of D-glucose from PNPG per min at pH 5.5 at 37°C.

### Characterization of the rAciHBGase II-(His)_6_ enzyme preparation

To evaluate the optimum reaction pH for α-glucosidase activity, the reaction mixture contained 0.05 ml of the r*Aci*HBGase II-(His)_6_ preparation, 0.05 ml of 5 mM PNPG and 0.1 ml of Briton-Robinson buffer (10 mM acetic acid, 10 mM phosphoric acid and 10 mM boric acid) at the desired pH (range of 3.0–7.5). The reaction mixture was incubated at 37°C for 10 min, and then stopped and assayed as above. For evaluation of the pH stability, the r*Aci*HBGase II-(His)_6_ preparation was prepared in Briton-Robinson buffer at the desired pH (range 3.0–12.0) and then stored at 4°C for 24 h before being adjusted to the previously determined optimal pH and assayed for α-glucosidase activity as above. The α-glucosidase activity was expressed as the% of the residual activity compared to the control sample that was not pretreated at 4°C for 24 h.

To evaluate the optimum reaction temperature for α-glucosidase activity, the reaction mixture was prepared as above except that the reaction was performed at pH 5.5 and at 25, 30, 35, 40, 45, 50, 55, 60 or 70°C. Moreover, to determine the thermal stability of the enzyme, the r*Aci*HBGase II-(His)_6_ preparation was prepared in 0.1 M sodium phosphate buffer containing 0.05% (v/v) Triton X-100 at pH 5.5 and kept at the indicated temperature (range 4–85°C) for 15 min before then being assayed for α-glucosidase activity as above. In each experiment, three independent repeats were done.

### r*Aci*HBGase II-(His)_6_ enzyme kinetics

To determine the enzyme’s substrate specificity, the α-glucosidase activity was determined as above except that the substrate used was varied from maltose, maltotriose, maltotetraose, isomaltose, sucrose and soluble starch at the indicated concentrations (*s*) of 0–60 mM for maltose and maltotriose, 0–30 mM for maltotetraose, 0–100 mM for isomaltose, 0–25 mM for PNPG, 0–20 mM for sucrose and 0–40 mM for soluble starch. In addition, for the non-PNPG substrates, the α-glucosidase activity was determined in terms of the amount of glucose liberated. To this end 0.1 ml of the reaction mixture was mixed with 0.2 ml of glucose assay reagent (Sigma) and incubated at 37°C for 30 min. Then, the reaction was stopped by the addition of 0.2 ml of 12 N H_2_SO_4_ and the absorbance at 540 nm was measured. In each experiment, three independent repeats were done. The amount of glucose liberated from the substrate was determined by the glucose oxidase-peroxidase method (glucose assay kit, Sigma) as in Eq. (1):

(1) $$\mathrm{Glucose}\;\left(\mathrm{mg}\right)=\left[\left(A_{540}\mathrm{of}\;\mathrm{test}\right)\left(\mathrm{standard}\;\mathrm{glucose}\;\mathrm{in}\;\mathrm{mg}\right)\right]/\left(A_{540}\mathrm{of}\;\mathrm{standard}\right)$$

## Results

### *AciHBGase II* transcript expression pattern

**Figure 1:**
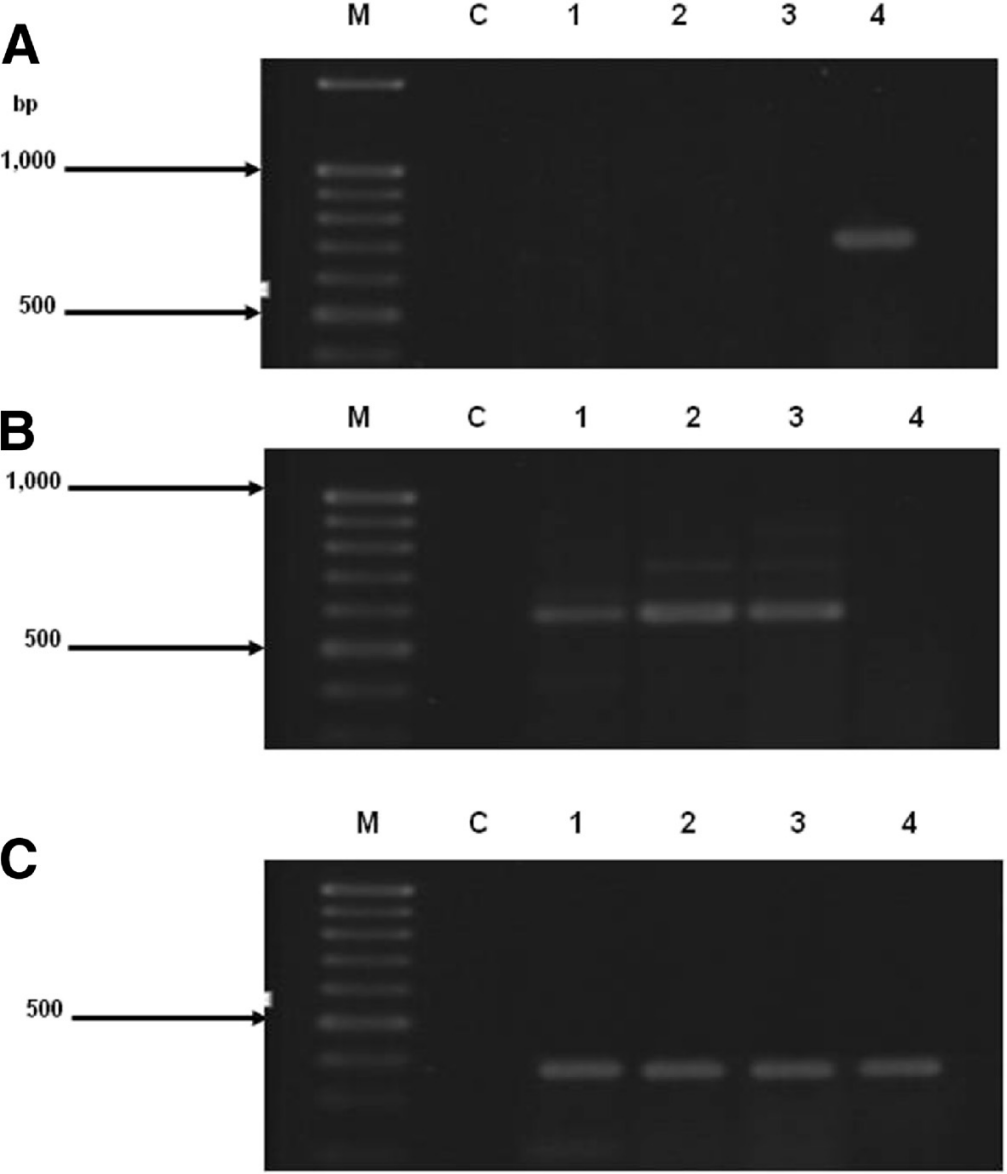
**Expression patterns of *AciHBGase I *and *II *in *A. cerana indica*.** The RT-PCR products amplified by the specific primer pairs for (**A**) *AciHBGase I* and (**B**) *AciHBGase II* with (**C**) the control profile of *28S rDNA* amplification. In each figure, lane M contained 100 bp DNA ladder marker, lane C was the negative control (no reverse transcriptase), and lanes 1-4 contained RT-PCR products from eggs, larvae, pupae and adult foragers, respectively. Gels shown are representative of those seen from three independent trials.

Comparison of the *AciHBGase* I and II transcript levels among egg, larvae, pupae and adult forager bees by semi-quantitative RT-PCR using primer pair 3_HBGase I and primer pair 2_HBGase II, respectively, was performed. Under the previously determined optimum condition for RT-PCR (data not shown), the highest expression of *AciHBGase I* transcripts was seen in adult forager bees since no detectable expression was found in eggs, larvae or pupae (Figure 1A), in keeping with the proposed role for this isoform in honey formation via hydrolysis of nectar derived sucrose in forager bees. In contrast, *AciHBGase II* transcripts were expressed in eggs and, especially, in larvae and pupae, but with no detectable expression in foragers (Figure 1B). As a template control, the expression of a fragment of the *28S rDNA* was observed in all samples at essentially the same level (Figure 1C). Of course this is the average transcript expression level across all larval, pupal and adult foraging bee tissues and so excludes any more subtle developmentally dependent tissue specific expression patterns.

### Full length cDNA sequence and its homology

**Figure 2:**
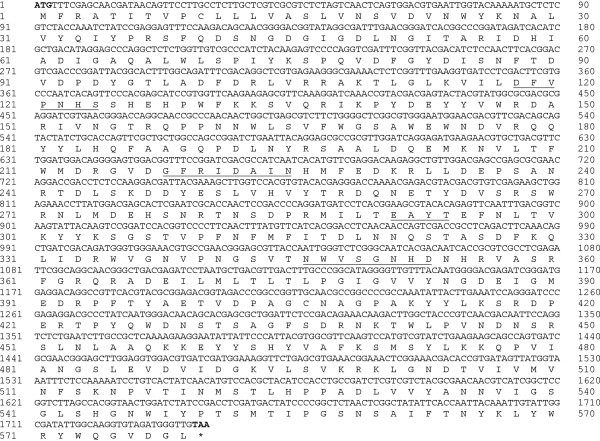
**The full-length *AciHBGase II *cDNA and deduced amino acid sequence of the encoded *Aci*HBGase II enzyme.** The predicted start (ATG) and stop (TAA) codons are shown in bold, giving a predicted open reading frame (ORF) of 1,740 bp encoding for a deduced 579 residue amino acid sequence. The conserved amino acid regions associated with the α-amylase family are underlined.

To obtain the full length cDNA nucleotide sequence of the *AciHBGase II* gene, primers were designed from various locations based upon the available *AmHBGase II* sequence (GenBank accession # NM_001040259). The expected RT-PCR products were successful amplified by the designed primers (Table 1) (data not shown), and then direct sequenced and assembled using the Clustal X program to obtain the full length consensus cDNA sequence of *AciHBGase II* at 1,740 bp (Figure 2). The sequence has been deposited at GenBank with accession code # JX468895. From the predicted ORF of the cDNA sequence the deduced encoded amino acid sequence of 579 amino acids was obtained (Figure 2). This predicted primary structure of *Aci*HBGase II showed the conserved regions associated with α–amylase family of enzymes [3]. Searching the NCBI GenBank database using the megaBLASTn algorithm (http://www.ncbi.nlm.nih.gov) revealed that the *AciHBGase II* cDNA nucleotide sequence was 90% identical to that of the *HBGase II* from the closely related *A. cerana japonica* bee (GenBank accession # NM_FJ752630.1). When the deduced amino acid sequence of *Aci*HBGase II was aligned to the amino acid sequences of homologs with similar annotated functions, a high amino acid sequence similarity was revealed (Figure 2).

### Expression of *rAci*HBGase II (as a C-terminal (His)_6_ tagged chimera)

**Figure 3:**
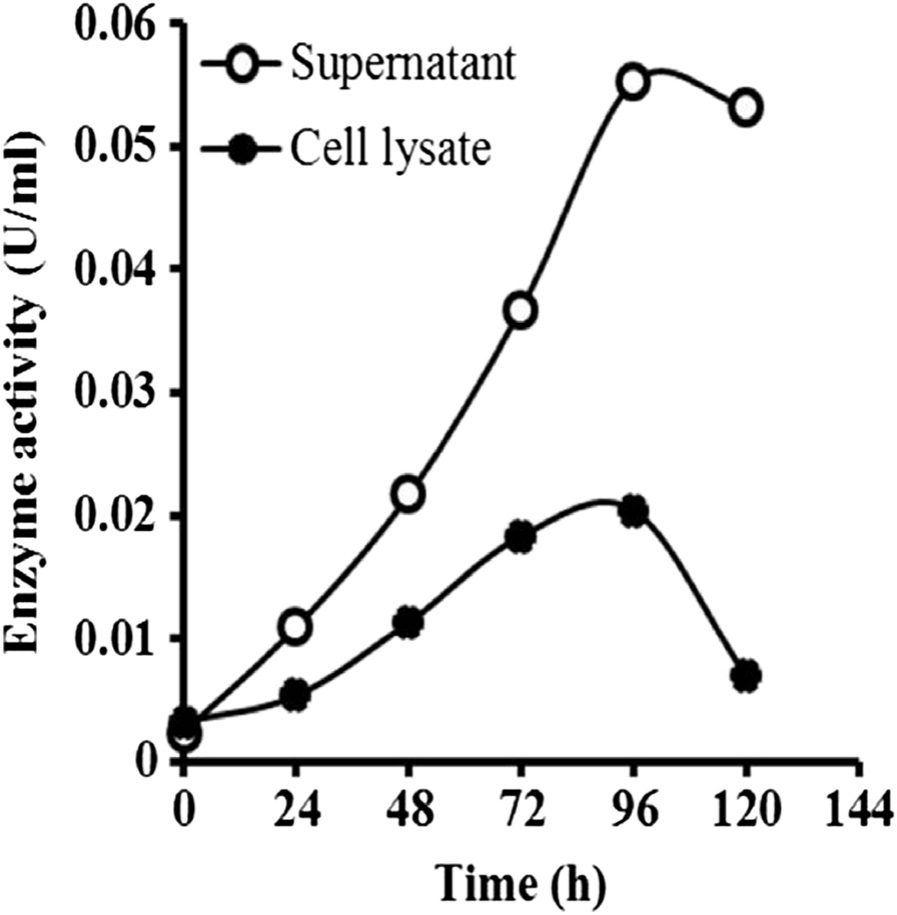
**Induction and expression of α-glucosidase activity.** α-Glucosidase activity levels (assumed to be from the r*Aci*HBGase II-(His)_6_ enzyme) in the culture medium (supernatant) and *P. pastoris* cell lysate after being induced by 0.5% (v/v) MeOH in 50 ml cultures.

The expression plasmid pPICZαA harboring the cDNA encoding the full length *AciHBGase II* was transformed into *P. pastoris* GS115. The selected transformant was cultured in BMMY medium (50 ml) and was induced by methanol (0–10% (v/v)) in order to express the r*Aci*HBGase II-(His)_6_ enzyme. The optimal methanol concentration, in terms of inducing the highest secretory enzyme activity, was found to be 0.5% (v/v) (data not shown). At this optimal methanol concentration (0.5% (v/v) added every 24 h), α- glucosidase activity (assumed to be due to the r*Aci*HBGase II-(His)_6_ enzyme) was found in both the cell lysate and supernatant and was maximal 96 h after induction, declining thereafter especially in the cell lysate (Figure 3). However, at all time points, including at the maximal expression time of 96 h after induction, a significantly greater α-glucosidase activity (2.5-fold) was obtained in the culture medium (supernatant) than in the cell lysate (~ 0.05 *vs*. ~ 0.02 U/ml, respectively). When the culture was scaled up to 1.2 l a 1.92-fold lower maximal α-glucosidase activity was obtained (0.026 U/ml) under otherwise the same conditions (data not shown).

### Purification of the r*Aci*HBGase II-(His)_6_ enzyme

**Figure 4:**
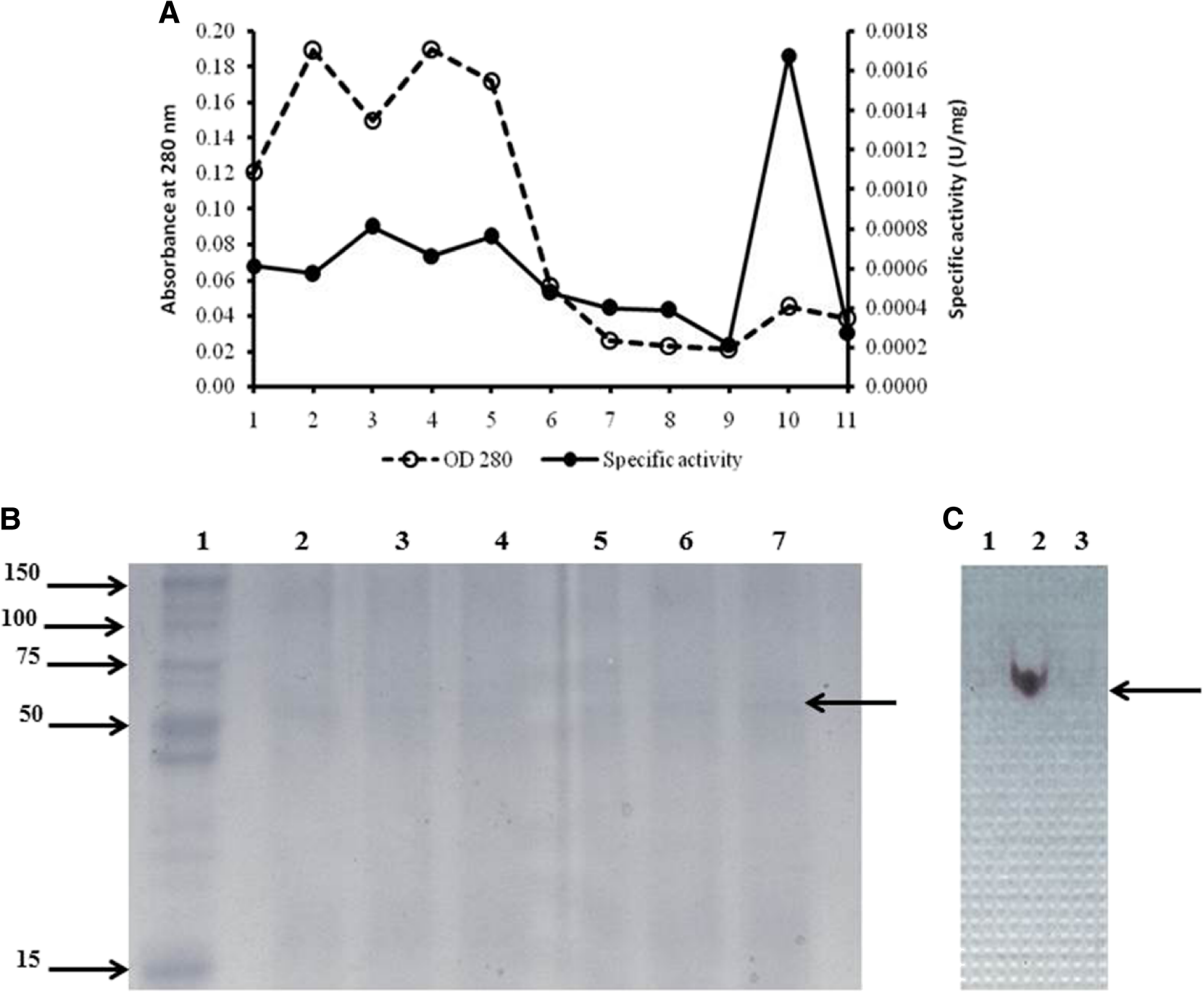
**Enrichment profile for the r*Aci*HBGase II–(His)**_**6 **_**protein by Histrap affinity column.** (**A**) Elution profile from the Histrap column. The washed fractions contained a high content of protein while eluted fraction # 10 presented the highest specific activity of HBGase II. (**B**) SDS-PAGE resolved and coomassie blue stained protein profile of selected fractions from the Histrap column elution. Lane 1: standard protein marker, lanes 2-6: contained 1 μg of protein in fractions # 2, 4, 6, 8 and 10, respectively. Lane 7 is a duplication of lane 6. (**C**) Glucosidase activity stain of the renatured SDS-PAGE resolved protein samples (Zymograph). Lanes 1-3, fractions # 9-11, respectively. Arrows in (**B**) and (**C**) indicate the ~ 73 kDa band.

**Figure 5:**
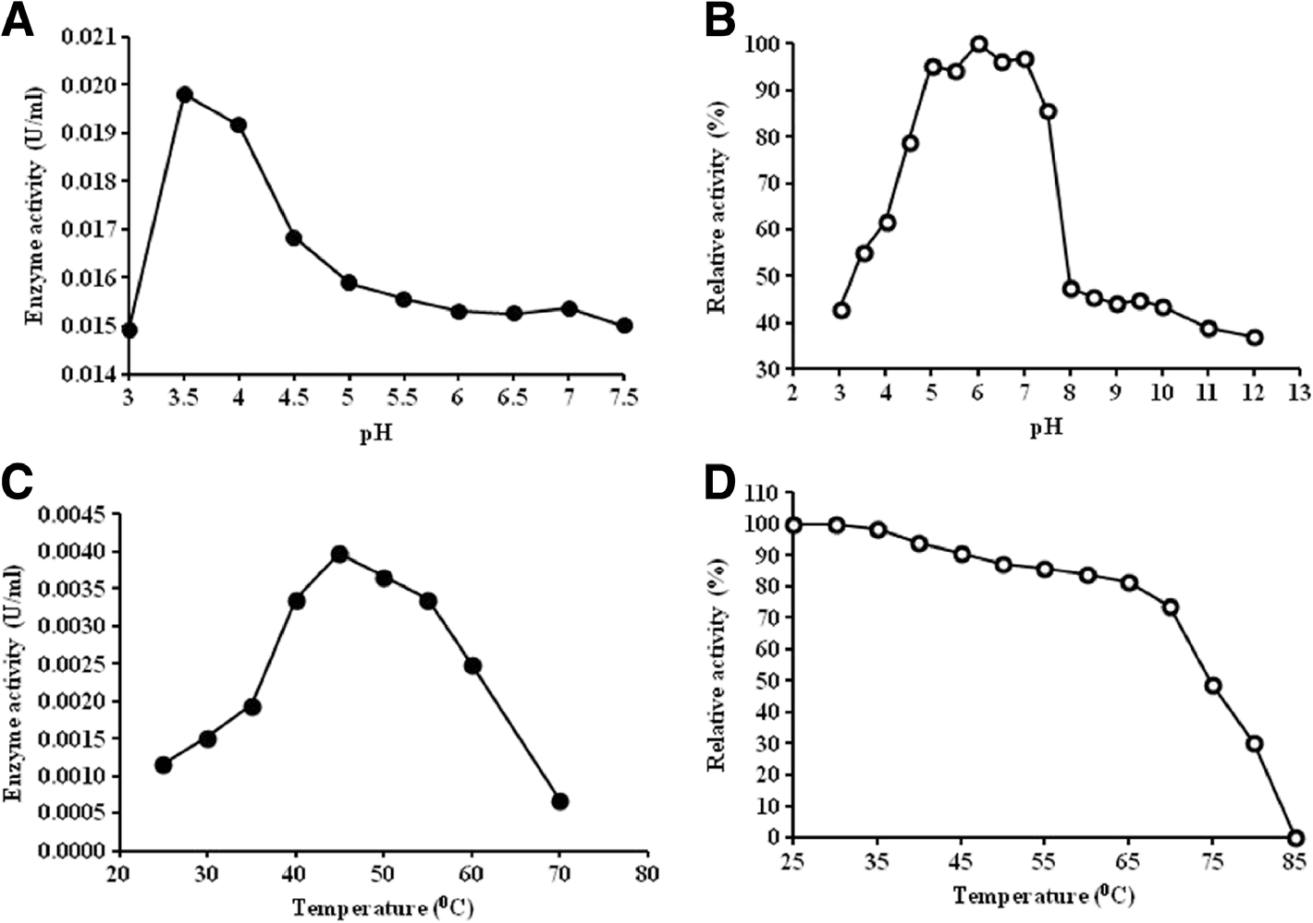
**The optimum conditions for α-glucosidase activity of the partially purified r*Aci*HBGase II–(His)**_**6 **_**enzyme.** The optimal (**A**) pH and (**C**) temperature and the (**B**) pH or (**D**) thermal stability.

After centrifugal filtration based concentration of the culture medium, the secreted r*Aci*HBGase II–(His)_6_ enzyme (1 ml) was further purified by Histrap affinity column chromatography (GE Healthcare). Eleven fractions (2 ml per fraction) were collected (kept at -20°C until use) and assayed for α-glucosidase activity using PNPG as the substrate. From the protein and enzyme activity elution profile (Figure 4A), fraction # 10 provided the highest enzyme specific activity (1.6 mU/mg). Although only a one step purification procedure was used, a single HBGase active band could be observed (Figures 4B and 5C). Thus, this fraction was used for further characterization. The enrichment data are summarized in Table 2.

**Table 2:** **Purification of the r*Aci*HBGase II-(His)**_**6 **_**enzyme**

**Procedure**	**TotalPrProtein (mg)**	**Total activity (U)**	**Specific activity (mU/mg)**^**a**^	**Yield (%)**	**Purification fold**
Negative control (Empty vector used)	3720	1.0	0.26	-	-
Supernatant (100 ml)	2326	2.6	0.11	100	1.0
Concentrated with Viva spin 20	151.5	0.20	1.3	7.69	1.20
Histrap column chromatography	11.25	0.018	1.6	0.69	1.45

^a^ mU = 10^-3^ U.

The rAciHBGase II-(His)_6_ enzyme was partially purified from 100 ml of a 1.2 liter culture of transformed *P. pastoris* after 96 h induction with 0.5% (v/v) methanol.

### Characterization of the r*Aci*HBGase II-(His)_6_ enzyme

#### Optimum pH and temperature

The effects of pH and temperature on the α-glucosidase activity of the partially purified r*Aci*HBGase II-(His)_6_ preparation were measured using PNPG as the substrate. The optimal activity was found at pH 3.5, although > 80% was observed at pH 3.5 – 5.0 (Figure 5A) and the enzyme was stable (> 90% residual activity) to a 24 h exposure at 4°C to pH in the range of 5.0 - 7.0 (Figure 5B). In addition, the optimal enzyme activity at pH 3.5 was found at 45°C, although 90% activity was found in the 40–55°C range (Figure 5C). The enzyme preparation was stabile (> 90% residual activity) to a 15 minute exposure of < 45°C, although > 80% residual activity was maintained after exposure to up to 65°C for 15 min, but decreased rapidly at higher temperatures (Figure 5D).

#### Substrate specificity

**Figure 6:**
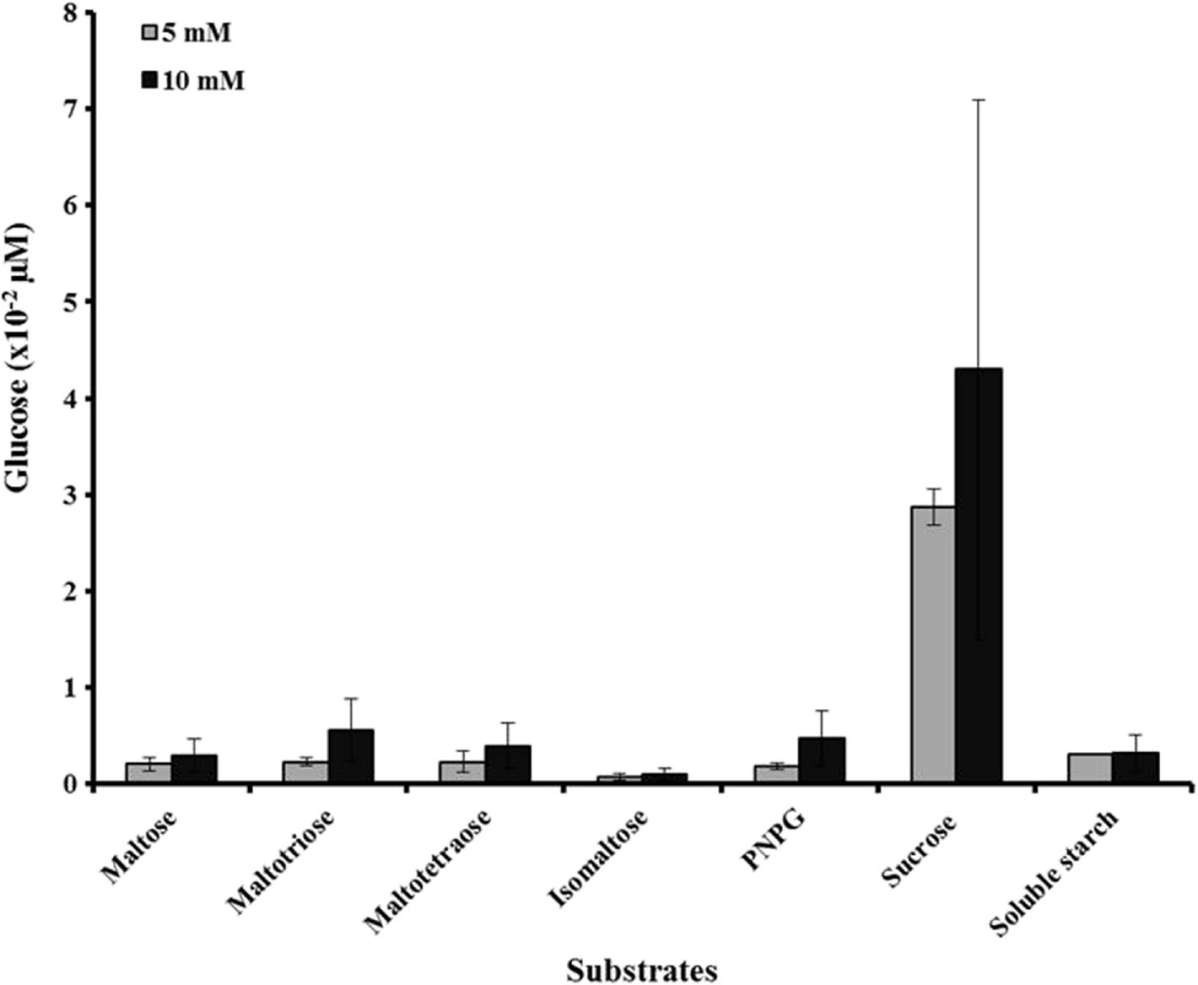
**Substrate specificity of the partially purified r*Aci*HBGase II-(His)**_**6 **_**preparation.** For PNPG each molecule of *p*-nitrophenol released is equated to one glucose molecule. Data are shown as the mean + 1 SD, and are derived from three replications.

**Figure 7:**
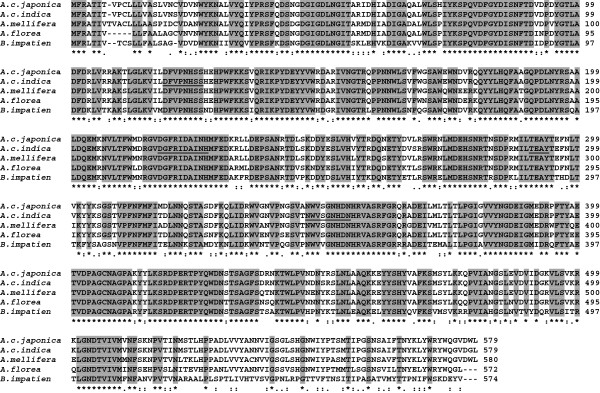
**Multiple amino acid sequence alignment of *Aci*HBGase II with other HBGase II homologs.** Shown are HBGase II sequences from *Apis cerana indica* (*Ac. indica*) in comparison with those from the Japanese honeybee *A. cerana japonica* (*Ac. japonica*, GenBank accession # ACN 63343.1), European honeybee *A. mellifera* (*A. mellifera*, GenBank accession # NP_001035349.1), predicted maltase 2-like of *Apis florea* (*A. florea*, GenBank accession # XP_003691502.1) and predicted maltase 2-like of *Bombus impatiens* (*B. impatien*, GenBank accession # XP_003493603.1). Sequences were aligned by the Clustal X program and the similarity across the aligned sequences is shown as identical (*), conserved (:) and semi-conserved (.). The conserved amino acid regions associated with the α-amylase family are underlined.

The substrate specificity of the partial purified r*Aci*HBGase II-(His)_6_ preparation was examined using seven different kinds of substrates, each at two different concentrations (5 and 10 mM), from which the best hydrolyzed substrate was found to be sucrose at both concentrations (Figure 6). Considering Figures 7 and 6 together, since the enzyme could hydrolyze sucrose very well, the deduced amino acid sequence of this enzyme was searched against the amino sequences in the NCBI GenBank database using the megaBLASTp algorithm, to check in particular for related sucrases or other enzymes that hydrolyze sucrose efficiently. The result showed that the enzyme was similar to β-fructosidase (sucrase) of *Thermotoga maritima* MSB8 (GenBank accession # O33833.1), sucrose-6-phosphate hydrolase of *Lactobacillus animalis* KCTC (GenBank accession # ZP_08548622.1), sucrose-6-phosphate hydrolase of *Lactobacillus ruminis* ATCC 25644 (GenBank accession # ZP_08079921.1) at 38%, 37% and 71% amino acid identity, respectively. This may also support the broad specificity of the enzyme.

#### Enzyme kinetics

When the substrate concentration (*s*) was plotted against the experimentally determined velocity (*v*) (Additional file 1: Figure S1), three possibilities could be observed. The first one, standard single-substrate Michaelis-Menten shape curves were observed for maltotriose, maltotetraose and sucrose. In contrast, for isomaltose, PNPG and soluble starch, since the curve decreased after attaining a maximum, polynomial shapes as the second one was revealed. For maltose, the plateau phase was not reached within the concentration ranges tested (0–60 mM). Thus, higher substrate concentrations must be used in order to establish the saturation [22].

Lineweaver-Burk plots (1/*s* versus 1/*v* plots) [23] revealed a linear regression for all the evaluated substrates (Additional file 2: Figure S2). The derived kinetic parameters (*K*_m_, *k*_0_ and V_max_) for the hydrolysis of those substrates are reported in Table 3, except for maltose since the reaction was clearly far off reaching a saturation in the concentration range tested (Additional file 1: Figure S1).

**Table 3:** **Kinetic parameters**^**a **^**for the hydrolysis of different substrates by the partial purified r*Aci*HBGase II-(His)**_**6 **_**enzyme**

**Substrate**	***K***_**m**_^**b**^	***k***_**0**_	***V***_**max**_^**c**^	***k***_**0**_**/*K***_**m**_
	**(mM)**	**(s**^**-1**^**)**	**(mg glucose/min/mg protein)**	**(mM**^**-1**^**·s**^**-1**^**)**
Maltotriose	26.7 ± 1.96	26.6 ± 0.88	3.94 ± 0.13	1.00
Maltotetraose	25.0 ± 3.10	2.84 ± 0.31	0.42 ± 0.04	0.11
Isomaltose	22.3 ± 3.49	5.2 ± 0.46	0.77 ± 0.07	0.23
Sucrose	15.3 ± 0.87	845.0 ± 0.00	125.0 ± 0.00	55.4
Soluble starch	22.0 ± 1.28	4.60 ± 0.14	0.68 ± 0.04	0.21
PNPG	4.20 ± 0.18	1.89 ± 0.11	0.28 ± 0.02	0.45

^a^Data are shown as the mean ± 1 SD and are derived from three independent repeats.

^b^The *K*_*m*_ value is the substrate concentration giving one half of the intrinsic *V*_max_.

^c^V_max_ was obtained from the calculated value as the glucose liberated from the reducing end residue of substrates.

Although the *K*_m_ values for maltotriose and maltotetraose were very similar, the *k*_0_ and V_max_ values were 9.4-fold larger for maltotriose than maltotetraose. The enzyme showed high activities towards PNPG and sucrose as substrates. The lowest *K*_m_ value was from PNPG (4.2 mM) although the V_max_ value was also the lowest (0.28 mg glucose/min/mg protein). This may indicate the enzyme could bind to PNPG very well leading to catalysis but that the reaction was not efficient or had a slow off rate. Whilst PNPG is not a biological substrate, such traits, if found for real substrates, would potentially be useful where some cellular compartments had only a low concentration of that sugar substrate. In the future, more experiments on determining the importance of a substrate presenting (i) low *K*_m_ and low V_max_ values, (ii) high *K*_m_ and low V_max_ values, (iii) low *K*_m_ and high V_max_ and (iv) high *K*_m_ and high V_max_ to cells should be performed.

## Discussion

The *AciHBGase III* transcript expression pattern [24] and nucleotide sequence have been reported previously [25]. Here we report that for the *AciHBGase II* isoform. Since some *AciHBGase II* transcript expression was observed in eggs and a higher expression level was found in larvae and pupae, but not in adult foraging bees (Figure 1), then *Aci*HBGase II is not likely to play a role in honey synthesis through the hydrolysis of sucrose from nectar in the honey crop [7], although we did not formerly test for expression levels in specific tissues including the hyopharangeal and salivary glands and honey crop, but rather screened whole adults. Nevertheless, it is more likely that *Aci*HBGase II is involved in other functions and possibly to do with the development of this honeybee. Hercovics [26] reported that endoplasmic reticulum (ER) - resident HBGase I and II played a sequential action on removing the terminal α-(1, 2)– linked and the two more internal α-(1,3)– linked glucose residues from post-modified glycoproteins in the ER. If HBGase is defective, it can lead to a negative effect on cellular functions [27].

The full length deduced open reading frame (ORFs) of the obtained *AciHBGase II* cDNA was 1,740 bp while the ORF of this gene homolog in *A. mellifera* was 1,743 bp [19], with the four highly conserved regions found in α–amylase family members being present. With respect to the nucleotide and predicted amino acid sequences, *Aci*HBGases can be classified into three isoforms (*Aci*HBGase I, II and III) as per the HBGases in the closely related *A*. *mellifera* and *A. cerana japonica* honey bees [19,28]. Different isoforms of enzymes can display marked differences in their kinetics and especially in their substrate specificities, as well as in the tissue or developmental stage dependent control of their expression levels or response to stimuli [29].

The secreted form of the r*Aci*HBGase II (as a chimeric protein with a C-terminal (His)_6_ tag) could be enriched by only a few purification steps (Figure 4 and Table 3). The partially purified r*Aci*HBGase II–(His)_6_ protein appeared to be active under relatively acidic conditions with a pH optima of 3.5, and with > 80% activity at pH 3.5–5.0, as well as being more acid-stabile than neutral to alkali. This is somewhat like that reported for the mannose 6-phosphate containing acid HBGase purified from bovine testes [30] and the human placental lysosomal acid HBGase that hydrolyzes glycogen to glucose [31]. As such it is potentially consistent with the proposed role of *Aci*HBGase II in the ER rather than in sucrose metabolism for honey production. Pompe disease, an autosomal recessive metabolic myopathy in the glycogen storage disease type II category, is caused by a deficiency of acid HBGase activity in humans. Thus, other than industrial enzyme production, heterogeneous expression of this gene in *P. pastoris* may be useful for this disease treatment. Furthermore, the optimum temperature for α-glucosidase activity of the r*Aci*HBGase II-(His)_6_ enzyme was at 45°C (with 80–90% residual activity at 40–55°C), which is much higher than that previously reported by Wu et al. [32], and higher than the physiological temperature of the bees. However, within extreme thermophiles, Nashiru et al. [33] reported a novel HBGase from *Thermus caldophilus* GK24 with an optimal temperature of 90°C, whilst the intracellular and extracellular HBGases from *G. toebii* strain E134 showed an optimum activity of 65°C and 70°C, respectively [34].

The enriched r*Aci*HBGase II-(His)_6_ enzyme showed a reasonably wide substrate specificity encompassing α-(1,1)-, α-(1,2)-, α-(1,4)- and α-(1,6)-glucosidic linkages, but rapidly hydrolyzed PNPG α-(1,6) and sucrose α-(1,2). Similar to the native *Am*HBGase II that displayed positive cooperativity to sucrose, turanose α-(1,3), kojibiose α-(1,2) and soluble starch α-(1,4) [10], the r*Aci*HBGase II-(His)_6_ enzyme hydrolyzed isomaltose and soluble starch but with different *K*_m_ values. This is expected since the substrate specificity of HBGases is reported to differ greatly with the enzyme source [35], one of the reasons for searching for new sources and thus isozymes with novel properties. Also, it may imply a difference in the recognition mechanism for allosteric ligands between enzymes or in the structures of the substrate-binding and catalytic sites of enzymes. Nevertheless, the difference in enzyme products could also be explained by different mechanisms employed by recombinant and native enzymes [36], and so it awaits the purification and characterization of the native *Aci*HBGase II for comparison.

The use of a mini-fermentor could be considered for scale-up experiments instead of culture in a shaken flask in order to improve the efficiency of product production and the level of expression of foreign protein in *P. pastoris*.

## Conclusion

We isolated the full length cDNA and predicted ORF of an α – glucosidase II from *A. cerana indica* (*AciHBGase II*), a gene whose transcript expression pattern was found to be restricted to the egg to pupal but not adult forager developmental stages. An expression plasmid containing the *AciHBGase II* gene was constructed and over-expressed in *P. pastoris*. The characteristics of the obtained r*Aci*HBGase II-(His)_6_ chimeric enzyme, its acid activity (optimal at pH 3.5 and > 80% activity at pH 3.5 – 5.0) and acid tolerance plus its activity at relatively high temperatures (45°C optimal and > 80% activity at 45–55°C) suggest it could be of interest for enzyme applications and in biotechnological processes when the conformational stability of the enzyme to heat and pH are required.

## Abbreviations

HBGase: α–glucosidase; *Aci*HBGase: α–glucosidase from *A. cerana indica*; *Am*HBGase: α–glucosidase from *A. mellifera*; (r)HBGase: Recombinant α–glucosidase; r*Aci*HBGase: Recombinant α-glucosidase from *A. cerana indica*; r*Aci*HBGase II-(His)_6_: r*Aci*HBGase as a C-terminal (His)_6_ tagged chimera.

## Competing interests

The authors declare that they have no competing interests.

## Authors’ contributions

JK and MK performed the experiments, analyzed and interpreted the data. CC contributed to the study design and supervision of the experiments and contributed in drafting the manuscript. UL, KK and AK contributed to the supervision of the experiments, help and discussion of the manuscript. All authors read the manuscript, contributed in correcting it and approved its final version.

## Supplementary Material

Additional file 1: Figure S1 Substrate (*s*) versus enzyme velocity (*v*) plots for the hydrolysis reaction with the partially purified r*Aci*HBGase II-(His)_6_ preparation. Shown are with the results for, (**A**) maltotriose (0–60 mM), (**B**) maltotetraose (0–30 mM), (**C**) isomaltose (0–100 mM), (**D**) PNPG (0–25 mM), (**E**) sucrose (0–20 mM), and (**F**) soluble starch (0–40 mM) as substrates. For PNPG each molecule of *p*-nitrophenol released is equated to one glucose molecule. Click here for file

Additional file 2: Figure S2 Lineweaver-Burk plots for the hydrolysis reaction with the partial purified r*Aci*HBGase II-(His)_6_ preparation. Substrates shown are (**A**) maltotriose (0–60 mM), (**B**) maltotetraose (0–30 mM), (**C**) isomaltose (0–100 mM), (**D**) PNPG (0–25 mM), (**E**) sucrose (0–20 mM), and (**F**) soluble starch (0–40 mM). For PNPG each molecule of *p*-nitrophenol released is equated to one glucose molecule. The linear regression coefficiemt (R^2^) and equation of the best for line shown are given in each panel. Click here for file
