# Supplementary figures and images for: Expression of a secretory α-glucosidase II from Apis cerana indica in Pichia pastoris and its characterization
Source: BMC Biotechnol. 2013 Feb 18;13:16. doi: 10.1186/1472-6750-13-16 (PMC3599193; doi:10.1186/1472-6750-13-16)

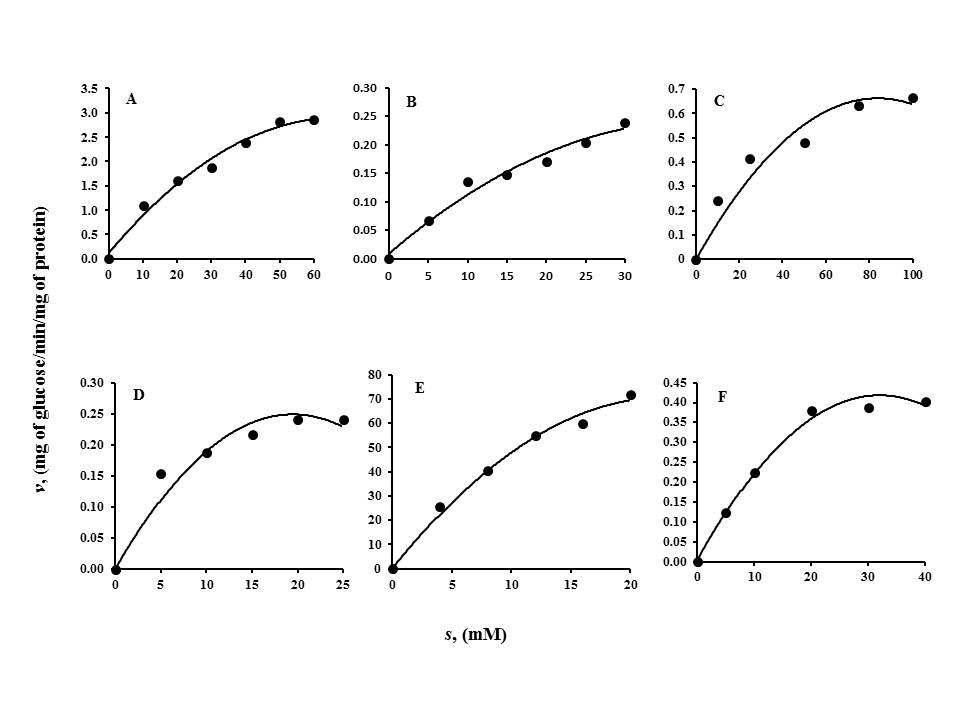

Supplement: Additional file 1: Figure S1 — Substrate (s) versus enzyme velocity (v) plots for the hydrolysis reaction with the partially purified rAciHBGase II-(His)6 preparation. Shown are with the results for, (A) maltotriose (0–60 mM), (B) maltotetraose (0–30 mM), (C) isomaltose (0–100 mM), (D) PNPG (0–25 mM), (E) sucrose (0–20 mM), and (F) soluble starch (0–40 mM) as substrates. For PNPG each molecule of p-nitrophenol released is equated to one glucose molecule. [file 1472-6750-13-16-S1.jpeg]

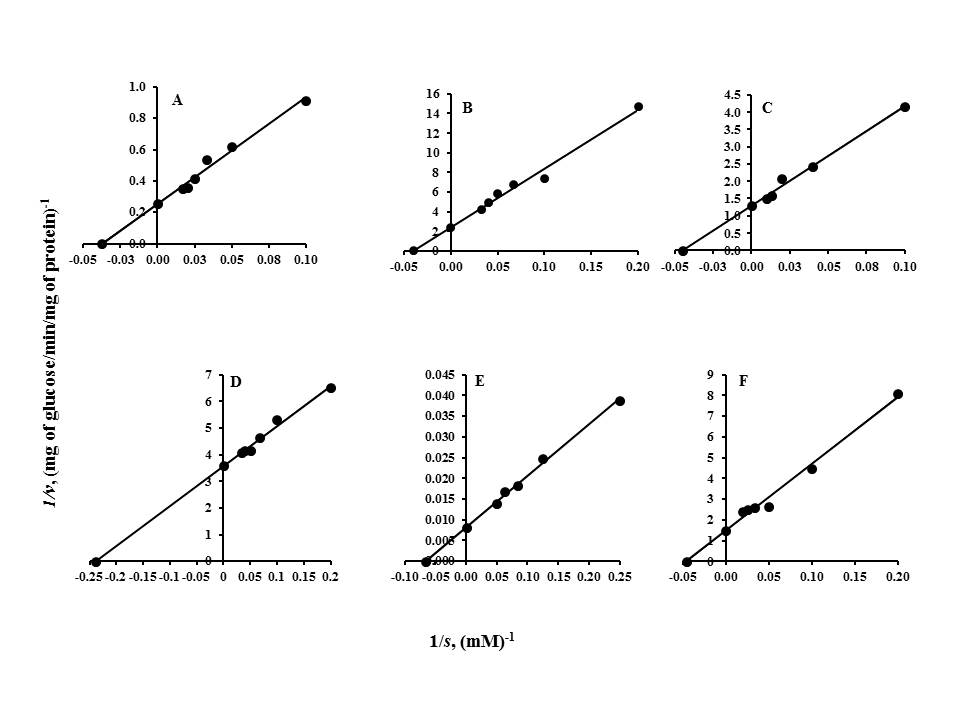

Supplement: Additional file 2: Figure S2 — Lineweaver-Burk plots for the hydrolysis reaction with the partial purified rAciHBGase II-(His)6 preparation. Substrates shown are (A) maltotriose (0–60 mM), (B) maltotetraose (0–30 mM), (C) isomaltose (0–100 mM), (D) PNPG (0–25 mM), (E) sucrose (0–20 mM), and (F) soluble starch (0–40 mM). For PNPG each molecule of p-nitrophenol released is equated to one glucose molecule. The linear regression coefficiemt (R2) and equation of the best for line shown are given in each panel. [file 1472-6750-13-16-S2.jpeg]
